# Transcriptional Regulation of Human *NANOG* by Alternate Promoters in Embryonic Stem Cells

**DOI:** 10.4172/2157-7633.S10-009

**Published:** 2012-08-24

**Authors:** Satyabrata Das, Snehalata Jena, Eun-Mi Kim, Nicholas Zavazava, Dana N. Levasseur

**Affiliations:** 1 Department of Internal Medicine, Roy J. and Lucille A. Carver College of Medicine, Iowa City, USA; 2 Programs in Genetics and Molecular and Cellular Biology, University of Iowa, Iowa City, Iowa 52242, USA

**Keywords:** *NANOG*, Alternative splicing, Alternate promoter, Pluripotency, Self-renewal, Differentiation, Stem cells

## Abstract

**Introduction:**

The potential of pluripotent stem cells to be used for cell therapy depends on a comprehensive understanding of the molecular mechanisms underlying their unique ability to specify cells of all germ layers while undergoing unlimited self-renewal. Alternative splicing and alternate promoter selection contribute to this mechanism by increasing the number of transcripts generated from a single gene locus and thus enabling expression of novel protein variants which may differ in their biological role. The homeodomain-containing transcription factor NANOG plays a critical role in maintaining the pluripotency of Embryonic Stem Cells (ESC). Therefore, a thorough understanding of the transcriptional regulation of the *NANOG* locus in ESCs is necessary.

**Methods:**

Regulatory footprints and transcription levels were identified for *NANOG* in human embryonic stem cells from data obtained using high-throughput sequencing methodologies. Quantitative real-time PCR following reverse transcription of RNA extracted human ESCs was used to validate the expression of transcripts from a region that extends upstream of the annotated *NANOG* transcriptional start. Promoter identification and characterization were performed using promoter reporter and electrophoretic mobility shift assays.

**Results:**

Transcriptionally active chromatin marking and transcription factor binding site enrichment were observed at a region upstream of the known transcriptional start site of *NANOG*. Expression of novel transcripts from this transcriptionally active region confirmed the existence of *NANOG* alternative splicing in human ESCs. We identified an alternate *NANOG* promoter of significant strength at this upstream region. We also discovered that *NANOG* autoregulates its expression by binding to its proximal downstream promoter.

**Conclusion:**

Our study reveals novel transcript expression from *NANOG* in human ESCs, indicating that alternative splicing increases the diversity of transcripts originating from the *NANOG* locus and that these transcripts are expressed by an alternate promoter. Alternative splicing and alternate promoter usage collaborate to regulate *NANOG*, enabling its function in the maintenance of ESCs.

## Introduction

Embryonic Stem Cells (ESCs), derived from the inner cell mass of the mammalian blastocyst, hold great promise for future therapeutic applications because of their unique ability to both proliferate indefinitely (self-renewal) in an undifferentiated state and retain the potential to give rise to every cell type in the body (pluripotency) [1–3]. Differentiated somatic cells can be reprogrammed into ESC-like Induced Pluripotent Stem cells (iPSCs) using various mixtures of pluripotency factors [4,5]. iPSCs provide a more attractive and patient-tailored platform for cell therapy, and proof-of-principle experiments in mice suggest genetic therapies using these cells are effective in ameliorating disease [6]. For iPSC based therapies to reach fruition, a more thorough understanding of the molecular mechanisms that govern the undifferentiated ESC state will be required.

Several recent studies using proteomics, RNA interference, loss of function screens, genome scale location analyses and transcriptome profiling have identified transcription factors that serve as the framework for maintaining ESC pluripotency and self-renewal [7–14]. The transcription factors NANOG, OCT4 and SOX2 constitute a core regulatory network which governs the stable expression of self-renewal factors while keeping gene differentiation programs suppressed in human and mouse ESCs [7,8,10,11,15]. Therefore, it is essential to understand the transcriptional properties of these core transcription factors to better enable controlled differentiation of pluripotent stem cells. Alternative splicing (AS) and alternate promoter usage adds another layer of regulatory control to the already complex process of pluripotency maintenance and differentiation. AS can vastly increase proteomic diversity by the addition or deletion of protein domains. These changes in coding sequence frequently lead to alterations in protein structure or subcellular localization, resulting in altered protein-protein interactions and biological outputs. AS mediated modifications of non-coding sequences can alter post-transcriptional regulation by mechanisms that include alternate 3’ untranslated region (UTR) usage and the associated revision of microRNA binding footprints [16]. Indeed ESCs express a large diversity of splice isoforms that implies switching from pluripotency to lineage commitment and differentiation involves AS and alternate promoter selection [17–24].

The homeobox-containing core transcription factor NANOG is specifically expressed in pluripotent cells of the mouse preimplantation embryo, embryonic germ (EG) cells and ESCs of murine [25–27] and human origin [28]. Disruption of NANOG leads to differentiation of ESCs into extraembryonic cell lineages in both murine [9,25,26] and human [29,30] ESCs. Overexpression of NANOG confers LIF (leukemia inhibitory factor)-independent self-renewal in mouse ESCs [26], and enables feeder-free propagation in human ESCs [31]. We recently reported an upstream extension of the mouse *Nanog* gene which enables production of the novel Nanog protein variants Nanog b and Nanog c that exhibit altered capacities for self-renewal and pluripotency in ESCs [23]. Another recent study has also reported a similar AS event in the human *NANOG* gene in embryonal carcinoma cells from an upstream region at the 5’ region, resulting in additional transcripts and a protein variant that initiates from a downstream methionine [32] and is the human ortholog of mouse Nanog c [23].

In the present study, we have verified the existence of novel alternate *NANOG* transcripts in human ESCs. We have identified a strong alternate promoter upstream of the novel transcripts using a neomycin resistance reporter assay that enables promoter strength to be assessed on chromatinized templates. The core transcription factors OCT4 and SOX2 have been shown to activate NANOG expression by binding to *cis*-regulatory elements in the proximal *NANOG* promoter [33], whereas Kruppel-like zinc-finger transcription factor KLF4 and the homeodomain containing transcription factor PBX1 also activate the *NANOG* promoter in cooperation with OCT4 and SOX2 [34]. We also demonstrate here that Nanog participates in positive autoregulation of its own proximal promoter.

## Materials and Methods

### Cell culture

Mouse ESC lines (CJ7 or J1) were maintained on gelatin-coated plates in a feeder-free condition as described previously in standard ESC media supplemented with LIF [35,36]. Human ESCs (H13, from WiCell) were cultured in DMEM/F12 medium supplemented with 20% Knockout Serum Replacement (GIBCO/BRL), 10 ng/ml bFGF, 1 mM GlutaMax, 50 U/ml penicillin and 50 µg/ml streptomycin, 1X nonessential amino acids and 100 µM 2-mercaptoethanol (Invitrogen) on top of γ-irradiated MEFs. Pluripotent ESCs were sorted from differentiated ESCs and MEFs using Pluripotent Stem Cell microbeads (Miltenyi Biotec).

### Plasmid construction

An EF1α-Flag-Biotin expression plasmid used previously [7] was adapted to analyze the ability of *NANOG* promoter fragments to drive the expression of a neomycin phosphotransferase coding sequence and impart neomycin resistance (Neo^R^). For this, the EF1α-Flag-Biotin sequence was removed and a Gateway recombination cassette (Invitrogen) was ligated in its place to generate a gateway-adapted plasmid. The Neo^R^ cassette was ligated downstream of the gateway cassette followed by a polyadenylation (polyA) signal from the *Nanog* 3’ UTR. Different fragments of the *NANOG* promoter were amplified from HEK/293T cell genomic DNA with attB site containing primers (Table 1). The PCR product was recombined into the pDONR221 entry vector by Gateway BP reaction and the promoter sequences were then transferred to the Gateway-adapted Neo^R^ destination vector by a Gateway LR reaction, followed by sequence verification.

For the luciferase reporter, the polyA sequence downstream of the firefly luciferase in the pGL3-basic plasmid (Promega) was replaced with the polyA signal from the *Nanog* 3’ UTR followed by Gateway adaptation of the plasmid by ligating a Gateway cassette into the MCS to generate the firefly luciferase destination vector. Different *NANOG* promoters were then PCR amplified with attB sites and recombined into the firefly luciferase destination vector by sequential BP-LR reactions as described above. Site-directed mutagenesis (Stratagene) was used to mutate the different transcription factor binding sites that were then verified by sequencing.

### Neomycin resistance assay

Linearized plasmids (5 µg each) containing different *NANOG* promoter fragments driving the Neo^R^ expression cassette were trasfected into 1×10^6^ J1 ESCs using Lipofectamine (Invitrogen) in 6-well plates. Transfected cells were selected with 200 µg/ml G-418 48 hr post-transfection for 8 days and stable colonies were counted. Assays were done in duplicate and were from at least two independent transfections.

### Luciferase reporter assay

The following constructs were cotransfected into 5×10^5^ CJ7 ESCs in 12-well plates: 2 µg of firefly luciferase reporter and 50 ng of the renilla luciferase vector (pRL-Null, Promega). 48 hrs post-transfection lysates were harvested for luciferase assays. Luciferase activity was measured by the Dual-Luciferase reporter assay system (Promega) using a BioTek Synergy 4 microplate reader. The firefly luciferase activity was normalized to the renilla activity to compensate for transfection variabilities in different wells. At a minimum, triplicate measurements were taken from two independent transfections.

### Electrophoretic mobility shift assay (EMSA)

Nuclear extracts were prepared from CJ7 ESCs as described previously [37,38]. Briefly, cells were harvested by trypsinization and washed twice with ice-cold phosphate-buffered saline and once with 5 pellet volumes of buffer A (20 mM Hepes pH 7.9, 10 mM KCl, 1 mM EDTA, 1 mM Na_3_VO_4_, 10% (vol/vol) glycerol freshly supplemented with 1 mM DTT, 1 mM PMSF, 1% protease inhibitor cocktail (Sigma)). Cell pellets were then incubated on ice for 10 min with 5 pellet volumes of buffer A and followed by lysis with 20 strokes in a Dounce homogenizer. Following lysis, pelleted nuclei were resuspended in 3 pellet volumes of buffer B (20 mM Hepes pH 7.9, 10 mM KCl, 350 mM NaCl, 1 mM EDTA, 1 mM Na_3_VO_4_, 20% (vol/vol) glycerol) freshly supplemented with 1 mM DTT, 1 mM PMSF, 1% protease inhibitor cocktail (Sigma)) and incubated at 4°C with rotation for 30 min. After that, the nuclear lysate was centrifuged and the supernatant containing the nuclear extract was used in the EMSA or stored at −80°C.

For EMSA, complementary oligonucleotides having an additional G at their respective 5’-ends were annealed in annealing buffer (10 mM Tris-Cl, pH 7.5, 1 mM EDTA, and 100 mM NaCl) and the resulting double-stranded (ds) oligonucleotides were end-filled with [γ-^32^P] dCTP using Roche Klenow for 30 minutes at 37°C. Following this, the radio-labeled ds-oligonucleotides were purified using Roche spin columns. For DNA binding reactions, 6 µg of nuclear extract was added to a 20 µl reaction containing 400 fM of radio-labeled oligonucleotide and 1 µg of poly(dIdC) (Roche) in the binding buffer (12 mM Hepes pH 7.9, 50 mM KCl, 1mM EDTA, 5% glycerol, 1mM DTT) and incubated on ice for 45 minutes. Where specified, 60 pM of unlabeled double-stranded competitor was included along with the radio-labeled oligonucleotide. Complexes were separated on pre-run 6% native polyacrylamide gels for 4 hrs at 150 V in 0.5X Tris-borate-EDTA. The gels were subsequently dried and autoradiography was performed.

### Sequence Analysis and bioinformatics

All Nanog DNA sequences are from the most recent genomic builds taken from the NCBI public database (July 2012 freeze). DNA alignments were done with CLUSTALW using the AlignX program from the VectorNTI suite. The DNAseI hypersensitivity (HS) peaks for the human H1 and H7 ESCs were obtained from ChIP-Seq data generated by the University of Washington ENCODE group [39]. The histone modification, RNA polymerase 2 and RBBP5 peaks were obtained for the H1 ESCs from the data generated by the Broad Institute/ MGH ENCODE group using ChIP-Seq [40]. The transcription level peaks were obtained for the H1 ESCs from data generated by the Wold Lab at Cal Tech, part of the ENCODE consortium assayed by high-throughput sequencing of polyA RNA. The vertebrate conservation measurement tracks were generated using multiz and other tools in the UCSC/Penn State Bioinformatics comparative genomics alignment pipeline. ChIP-Seq binding data for OCT4 and NANOG occupancy were obtained from Kunarso et al. [41] and the P300 ChIP-Seq data were from Lister et al. [42].

For data analysis and peak calling, Fastq files where downloaded from the NCBI SRA and aligned with bowtie 2-2.0.0-beta6 against the hg19 genome using default options. The aligned files where then combined using samtools merge to create one alignment file for each experiment (where multiple fastq files existed). Peak calling was then done with MACS 1.4.2 using default parameters except spacing was changed to 25 and wig file changed to single output. BigWig files were generated with the UCSC tool wigToBigWig.

### RNA extraction and quantitative real-time PCR

#### RT-PCR (qRT-PCR)

RNA was prepared from H13 human ESCs using the PARIS kit (Ambion) following the manufacturer’s instructions. An in-column DNAse digestion was performed to remove contaminating genomic DNA. cDNA was synthesized from 1 µg of RNA using the SSIII reverse transcriptase (Invitrogen) in a 20 µl total volume. cDNAs were diluted 40-fold and 5 µl of the dilute cDNA was used in a 25 µl SYBR Green reaction using a Bio-Rad CFX96. Technical replicates were represented from two independent biological replicates. Starting quantities were determined from a *GAPDH* standard curve.

## Results

### Transcriptional regulation of *NANOG* in human ESCs

**Figure 1:**
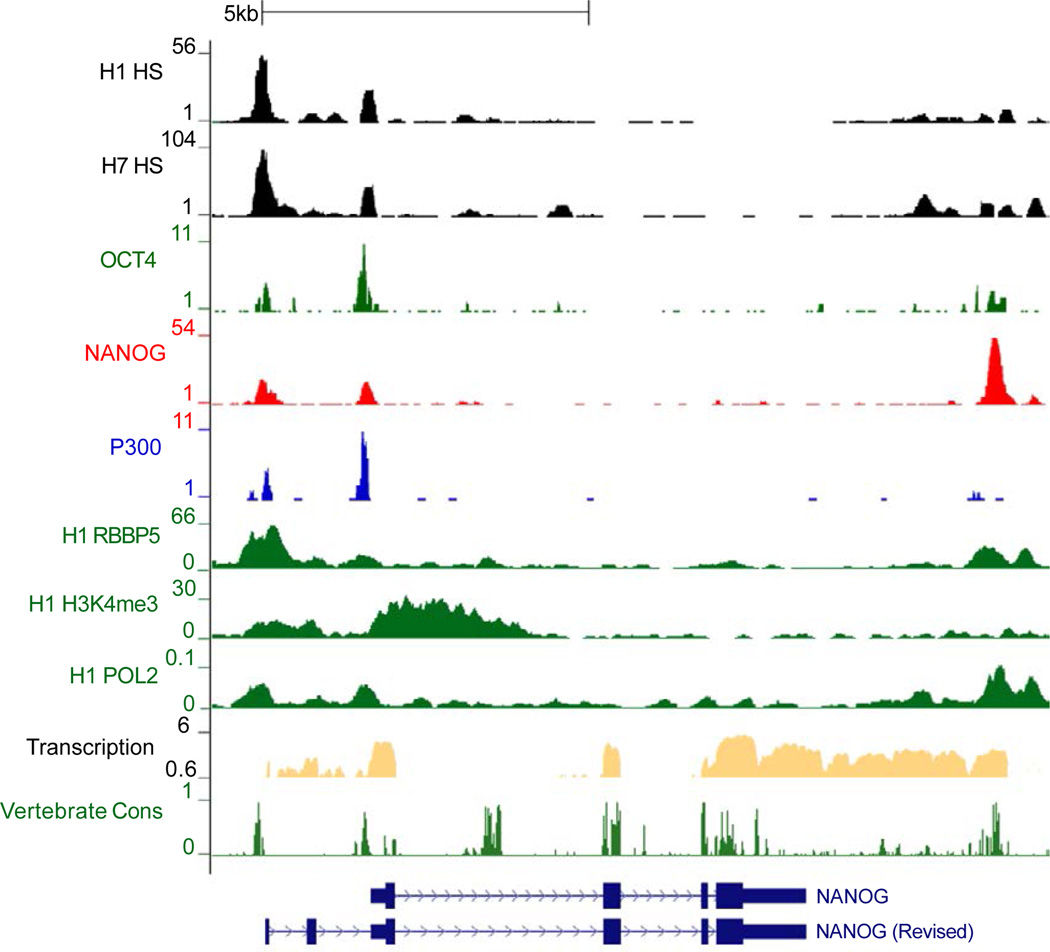
Transcriptional Regulation of NANOG in ESCs NANOG gene structure is shown at the bottom with the revised 5’ extended NANOG showing novel exons discovered recently in NTERA-2 cells [1]. NANOG regulatory regions are shown with DNAseI hypersensitivity peaks in H1 and H7 human ESCs. Chromatin occupancy profiles for ESC transcription factors (OCT4, NANOG), components of the basal transcriptional machinery (POL2 and p300) are shown at the NANOG locus in ESCs. The active chromatin mark H3 lysine 4 trimethylation (H3K4me3) profile and transcriptional expression levels generated by sequencing of polyA RNA are also depicted.

We have recently reported an extended gene structure of the mouse *Nanog* gene in ESCs [23]. Another study detailed the transcriptional properties of *NANOG* in a human embryonal carcinoma cell line, revealing additional exons at the 5’ end beyond the known gene structure [32]. A comprehensive survey of regulatory DNA within the NANOG locus had not previously been undertaken. We mined data from the ENCODE project and publically available databases to annotate transcriptional regulation of the *NANOG* gene in human ESCs [39–41]. DNAseI HS is an indicator of active *cis*-regulatory sequences. The data from two human ESC lines (H1 and H7) showed strong and identical DNAseI HS positioned 1.8 kb upstream from the known transcriptional start site (TSS) (Figure 1), whereas a less prominent HS was observed near the TSS. Significant binding enrichment for the basal transcriptional machinery elements represented by RNA polymerase II (POL2), P300 and the transcriptionally permissive chromatin mark histone H3 lysine 4 trimethylation (H3K4me3) were also observed at the −1.8 kb region and the known TSS (Figure 1). Binding enrichment for RBBP5, a component of the histone methyltransferase complex was also observed at both regions that were marked by H3K4me3. The ESC core transcription factors OCT4 and NANOG are known to bind the NANOG promoter, and both OCT4 and NANOG binding peaks were observed from the ChIP-Seq data at the −1.8 kb region and at the known TSS of *NANOG*. Transcription levels determined by sequencing of polyA RNA also demonstrate active gene expression at the known TSS and the - 1.8 kb region in human ESCs (Figure 1). These data, along with the already reported extended gene structure of *NANOG* in embryonal carcinoma cells, suggest the existence of a similar extension of the *NANOG* gene in human ESCs that approximates what we have observed for the murine *Nanog* gene [23].

**Figure 2:**
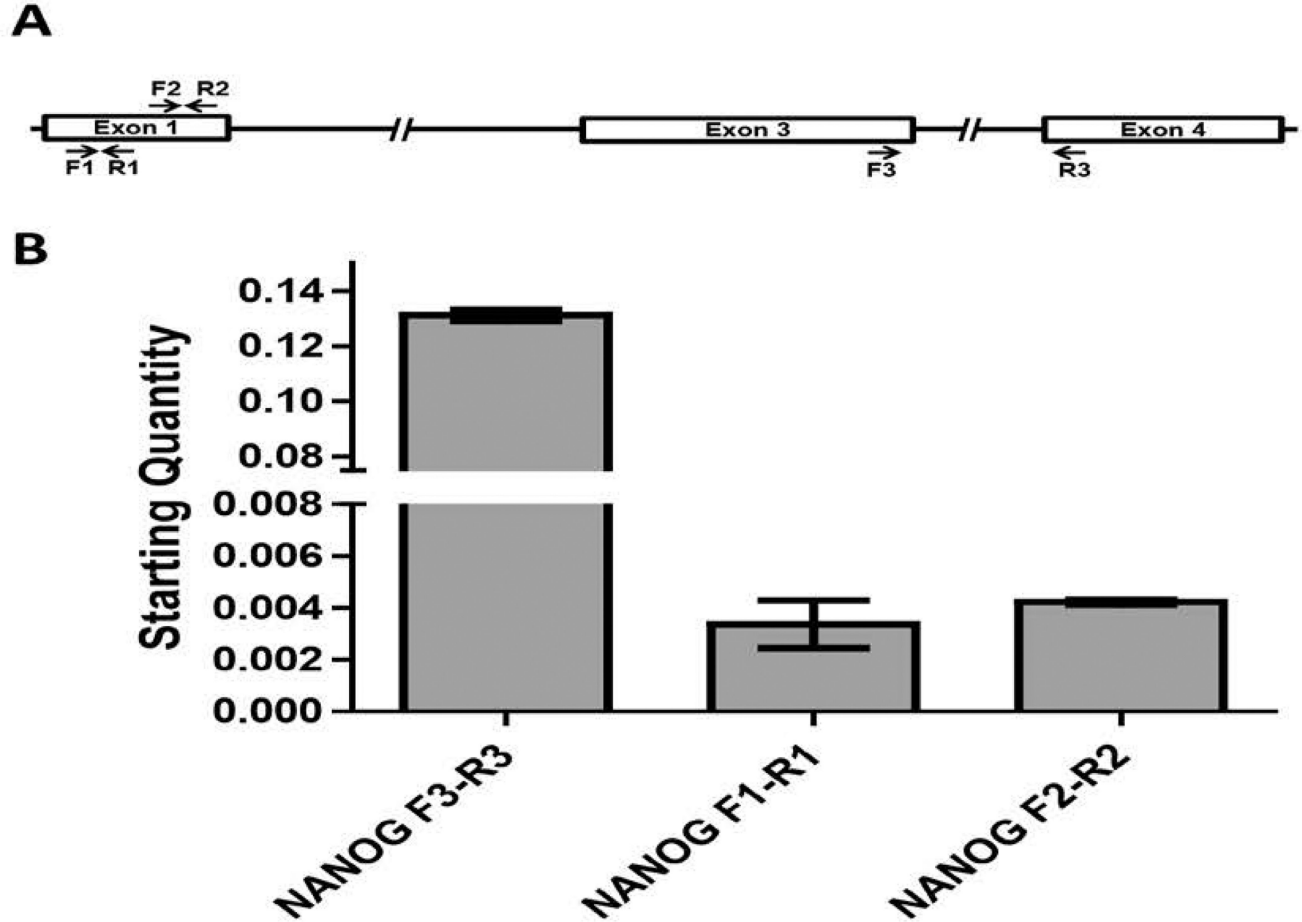
Novel *NANOG* transcripts in human ESCs (A) A schematic of the updated NANOG gene structure showing the location of the two primer pairs used to detect the exon 1 originating transcripts. The F3-R3 primer pair hybridizing to exons 3 and 4, respectively, was used to measure the total expression levels of total NANOG. qRT-PCR was used to measure the expression levels of total and novel *NANOG* transcripts initiating from the upstream exon 1 using RNA isolated from human H13 ESCs. The expression of upstream *NANOG* transcription is verified by two independent primer pairs. Levels shown are relative to a *GAPDH* control.

To confirm the existence of NANOG transcripts originating from the upstream exons in human ESCs as reported in NTERA-2 cells [32], we performed qRT-PCR using RNA from human H13 ESCs. cDNA was synthesized by reverse transcription using oligo (dT) primers to represent fully processed mRNA and qRT-PCR was performed. For detection of transcripts originating from the upstream region, primers were designed in exon 1 (see revised *NANOG* gene structure suggested by Eberle et al. [32] and shown in Figure 1) and to compare against total NANOG transcript levels, primers were designed spanning the junction of exon 3 and exon 4 (Figure 2A). The qRT-PCR results confirmed the presence of *NANOG* transcripts originating from the upstream region (Figure 2). As we had observed previously in mouse ESCs, the stoichiometric ratio was skewed toward transcripts from the downstream TSS. This was verified by two independent primer pairs (Figure 2).

### Alternate promoters regulate the expression of NANOG

**Figure 3:**
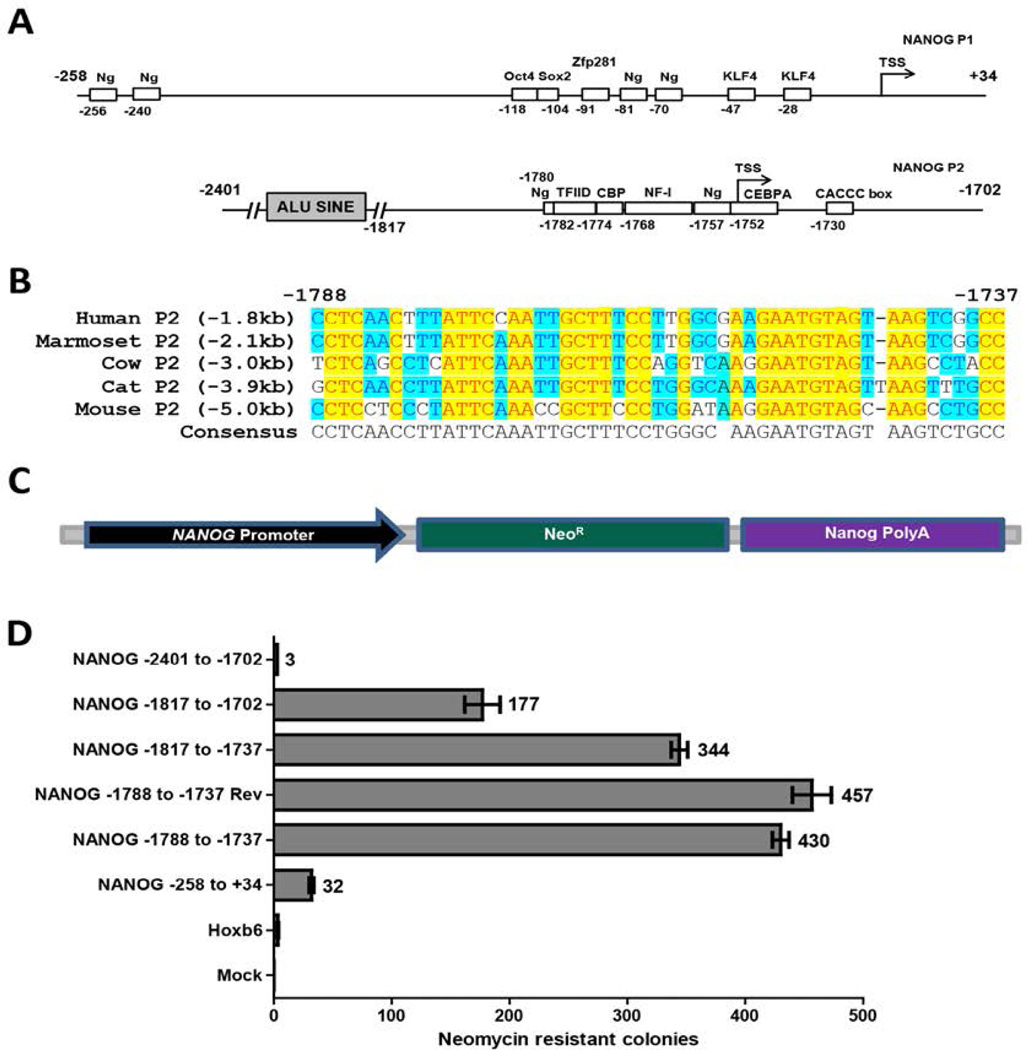
Neomycin resistance assay reveals alternate *NANOG* promoters in ESCs (A) Schematic of the two NANOG promoters P1 (proximal promoter, upper) and P2 (distal promoter, below) showing the transcription factor binding sites in rectangular boxes with their respective positions to the TSS. KLF4, OCT4 and SOX2 are validated sites, whereas the NANOG binding sites are predicted by the presence of the tetramer ATTA/C. P2 shows transcription factor binding sites that are predicted by TRANSFAC. (B) Alignment of the upstream P2 promoter in mouse, cat, cow, marmoset and human showing that the *NANOG* P2 is moving closer to the TSS across evolutionary time. (C) A schematic of the plasmid showing the design used for the neomycin resistance assay. (D) Neomycin resistant colonies produced by the respective promoters are plotted. Control cells were mock-transfected and the *Hoxb6* promoter was used as a negative control as it is not active in ESCs.

The existence of *NANOG* transcripts originating from a region upstream of the known TSS implied that this location must house a second promoter. The RNA polymerase II ChIP-Seq data (Figure 1) also revealed binding peaks at the upstream region suggesting RNA polymerase II recruitment to the upstream promoter. To explore and identify the second promoter, we amplified human genomic DNA regions upstream *of NANOG* exon 1 which is highly conserved among vertebrate sequences analyzed (Figure 3B). Different DNA fragments of the NANOG upstream region were used as promoters to drive expression of the neomycin resistance (Neo^R^) gene and form colonies in a chromatinized stable transfection assay that we employed previously to analyze murine Nanog regulatory elements [36]. The proximal promoter (−258 to +34) was used to compare the strength of the two promoter regions. The human *NANOG* proximal promoter has been shown to be highly active in mouse ESCs [33]; and unlike human ESCs, these cells are amenable to the Neo^R^ single cell colony assay. For these reasons, we performed the Neo^R^ assay in mouse J1 ESCs. High numbers of neomycin resistant colonies were obtained from cells transfected with either the proximal (P1) or the distal (P2) promoters in comparison to cells transfected with a *Hoxb6* promoter containing plasmid (Figure 3C and 3D). These results disclose a novel promoter (P2) upstream of the recently discovered exon 1 [32]. Although the proximal P1 promoter produced a significant number of neomycin resistant colonies in comparison to the control; surprisingly, P2 is much stronger than P1 in this assay. A minimal promoter consisting of the sequence between −1788 to −1737 produced the highest number of neomycin-resistant colonies. Analysis of this sequence by TRANSFAC predicted an abundance of transcription factor binding elements, including TFIID, CBP, CEBP alpha and NF-I in this short region (Figure 3A). This minimal promoter also has two well conserved predicted Nanog binding sites that are likely candidates for the significant NANOG occupancy we observe at this region. Additional DNA sequences at either end of the minimal promoter diminished the activation capacity of the minimal promoter. Inclusion of the Alu SINE (short interspersed element) upstream (*NANOG* −2401 to −1702) of the minimal promoter completely abolished the activation potential of the promoter revealing the inhibitory capacity of the SINE. Additionally, the minimal P2 promoter (−1788 to −1737) was orientation-independent as the sequence in reverse orientation also produced similar number of colonies. This orientation independence explains why the mouse counterpart of this regulatory element may have been categorized previously as an enhancer by our group and others [35,43,44]. These data reveal a novel and highly active promoter in ESCs which regulates the expression of the upstream *NANOG* transcripts.

### Nanog regulates the proximal promoter in ESCs

**Figure 4:**
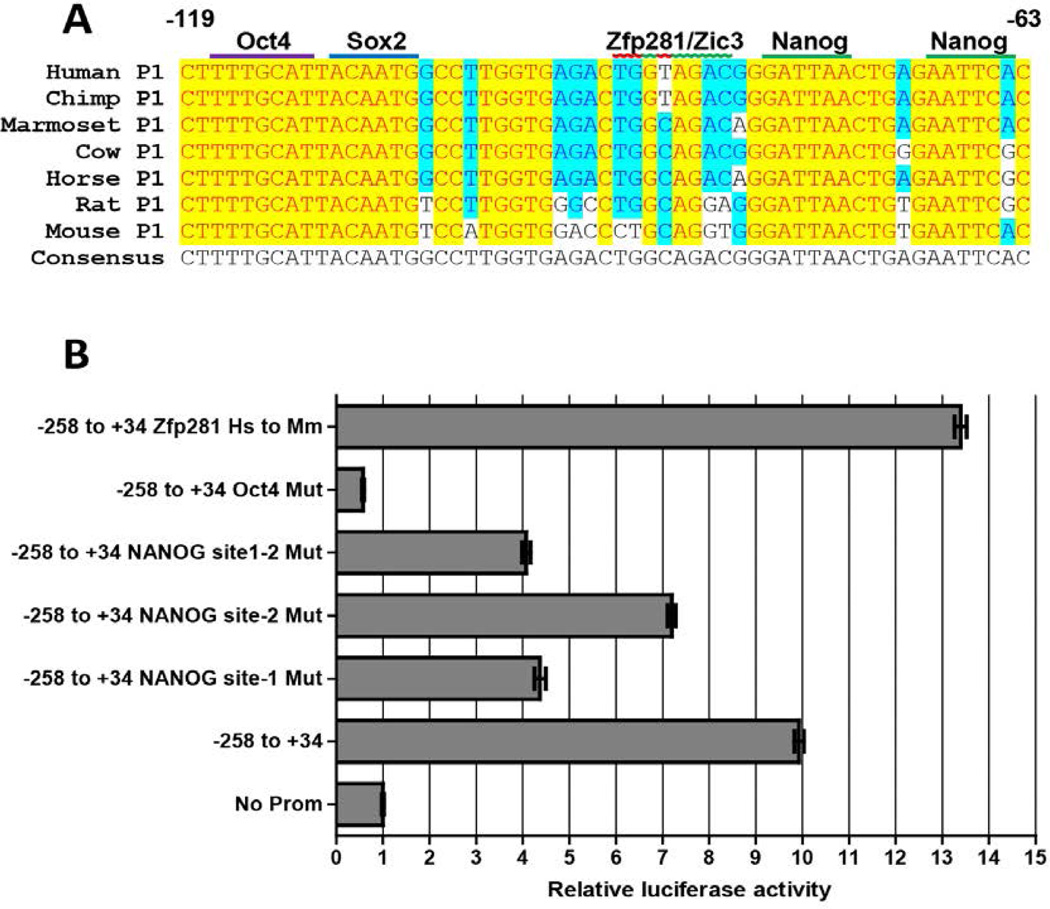
Nanog autoregulation of promoter P1 (A) An alignment of *NANOG* sequences from seven vertebrates in the region −119 to −63 relative to the human TSS. Oct4, Sox2 and the Nanog binding sites are perfectly conserved and indicated by solid bars, whereas the Zfp281/Zic3 site by a wavy line. The human (Hs) Zfp281/Zic3 site was mutagenized to produce a consensus Zic3/Zfp281 mouse site (Mm) by mutating CTGGTAG to CCTGCAG. (B) Luciferase assay was performed in CJ7 ESCs. Cells were transfected with the *NANOG* promoter reporter constructs (left) and analyzed for promoter activity. The Oct4 binding site was mutated GCAT to AACC and used as a positive control to show ablation of promoter activity. The Nanog binding sites were mutated from ATT to CCG at the conserved tetramer ATTA/C. Firefly luciferase expression levels were normalized to the luciferase activity of internal Renilla control. The no promoter containing plasmid (No Prom) was used as the internal control and its activity was normalized to 1. Data presented are the mean ± SEM of triplicates from one of two independent experiments.

Among the ESC core transcription factor triad it is well established that Oct4 and Sox2 are involved in the transcriptional regulation of Nanog [33,45,46]; however, the contribution Nanog makes in the regulation of its own promoter remains unexplored. To determine if Nanog employs an autoregulatory mechanism to control its expression we scanned the *NANOG* P1 for the presence of the most conserved tetramer ATTA/C reported to be present at the center of Nanog binding sites [15,25] and identified five such sites inside the P1 sequence (−258 to +34), including one that bridged the sense strand of the Oct4-Sox2 tandem binding site. We wish to note here that a well evolutionarily conserved consensus TATA box (TATAAA) [47] exists at +3 bp according to the Genbank TSS (NM 024865.2, July 2012) that was annotated from NTERA-2 expressed sequence tags, including the cDNA originally deposited by Shinya Yamanaka (Accession number AK022643) [48]. The mouse and cow TSS, determined from ESCs and blastocysts, respectively, is placed at the exact expected position 32 bp downstream of this TATA box (see NM 028016.2, June 2012 and NM 001025344.1, March 2012). We designed our P1 promoter based on this information and anticipate that future determination of the TSS in human ESCs using 5’ amplification of cDNA ends (RACE) will result in the updating of our P1 coordinates to −291 to +1. We employed a luciferase reporter assay to evaluate the contribution of presumptive NANOG ATTA/C binding sites in the regulation of P1 activity in CJ7 ESCs. In all predicted Nanog binding sites, ATT was mutated to CCG in the conserved tetramer to ablate Nanog binding. To disrupt the site hidden inside Oct4-Sox2 (ttttgcatTacaatg, predicted Nanog binding site underlined), only the T spacer nucleotide joining the Oct4 and Sox2 binding sites was mutated to A to ensure that the Oct4 and Sox2 binding sites remained functional. *NANOG* P1 induced high-levels of luciferase expression. Out of the five predicted Nanog binding sites tested, mutations at sites between −256 to −249, −240 to −233 and the site between Oct4-Sox2 binding had no effect on luciferase expression (data not shown). However, mutation of the sites at −81 to −74 (Nanog site-1) and −60 to −63 (Nanog site-2) resulted in 56% and 22% reductions in luciferase expression (Figure 4B) compared to the intact P1. Mutating both the sites resulted in a 59% reduction in the P1 luciferase activity. A sequence alignment of this *Nanog* promoter region from seven vertebrates shows that along with the Oct4-Sox2 binding *cis*-regulatory elements, the Nanog binding sites are also perfectly conserved throughout mammalian evolution (Figure 4A).

### Nanog binds to the *NANOG* proximal promoter

**Figure 5:**
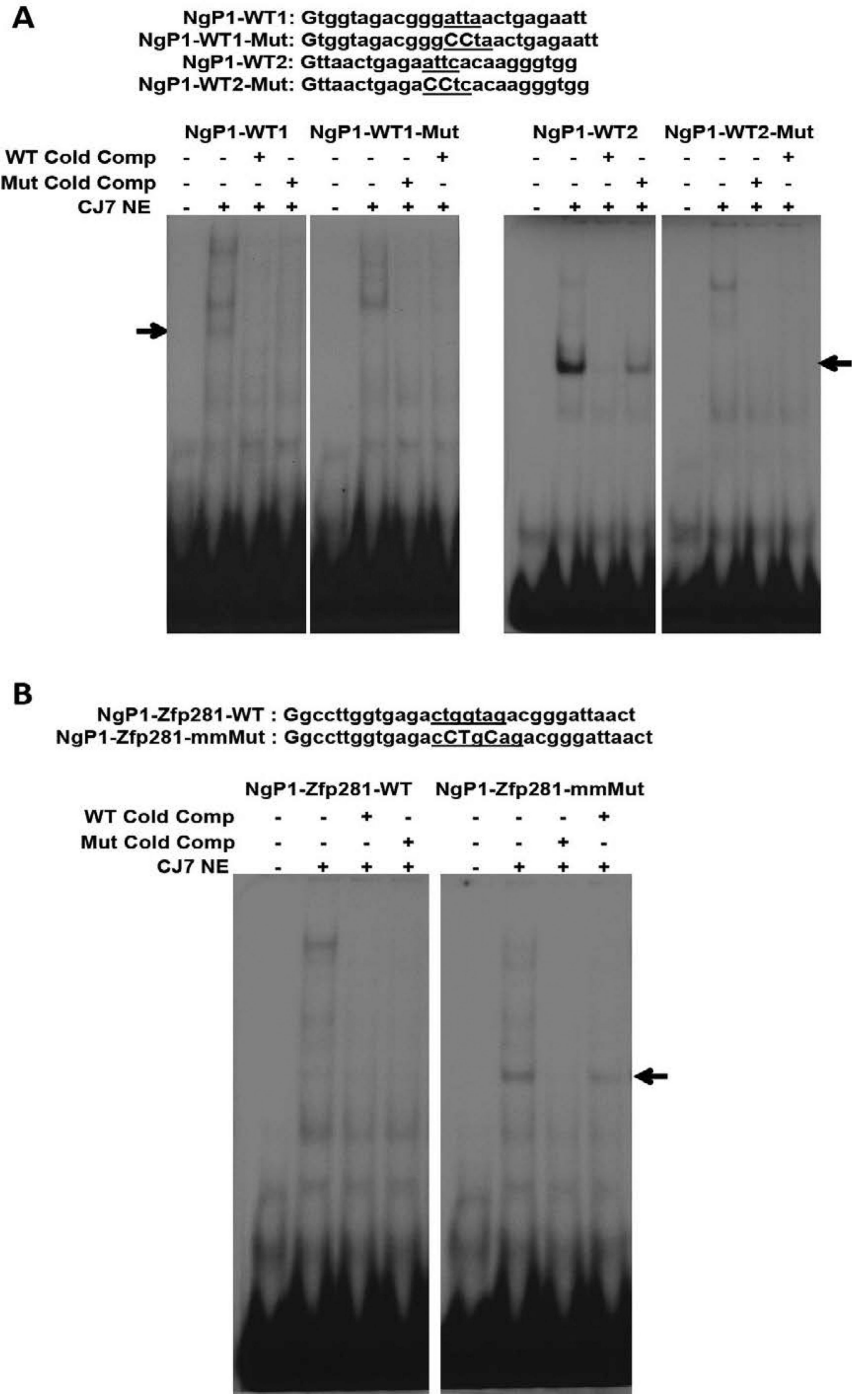
Nanog binds to the Nanog-binding sites in the human *NANOG* promoter (A) Sequence of the Nanog motif (underlined) containing *NANOG* proximal promoter oligonucleotides used as probe. Mutant (Mut) oligonucleotides have their binding motifs disrupted as shown in capital letters. Oligonucleotides were annealed to their antisense strands and labeled with [γ-^32^P] dCTP using Klenow by filling the G at both ends of the probe. Unlabeled oligonucleotides of the WT and Mut sequences were used for competitive binding. Binding was tested using nuclear extracts of CJ7 ES cells. Protein-DNA complexes of the resulting from Nanog binding is shown by arrow mark in the WT probes, whereas these shifts were not observed with the mutant probes. Unlabeled mutant oligonucleotides were also not able to compete off the binding resulting from the WT probes. (B) Sequence of the Zfp281 motif (underlined) restored to the mouse sequence of the *Nanog* promoter. Shift observed by incubation of the Zfp281-site restored probe incubation with CJ7 nuclear extract (indicated by arrow) was not observed by the human WT probe. The unlabeled human WT probe was also unable to compete off the shift resulting from the mutant probe.

After observing the reduction in *NANOG* P1 activity resulting from Nanog binding site mutagenesis we performed EMSAs to verify the ability of Nanog to bind these sites *in vitro*. Two different DNA fragments encompassing the Nanog binding sites were synthesized (Figure 5A) and radio-labeled. Nuclear extracts prepared from CJ7 ESCs resulted in shifts with both the probes. However, the binding was stronger at the Nanog site-2 containing probe in comparison to the site-1 (Figure 5A). These bindings were competed off with excess unlabeled oligonucleotide. In contrast, oligonucleotides with the mutated Nanog binding site failed to compete as expected. Incubation of the nuclear extracts with mutant probes also did not result in the shift obtained by wild-type probes. These data show that the Nanog binding sites recruit Nanog and imply that this recruitment contributes to the activation of *NANOG* expression that we observe.

### Zfp281 binding site in the *NANOG* P1 promoter is lost during evolution

Analysis of the Nanog P1 promoter sequence alignment revealed that the mouse Zfp281 binding site [49] is not well conserved in the human *NANOG* P1 (Figure 4A). This site is also bound by the pluripotency factor Zic3 as demonstrated by Nanog promoter activation in ESCs [50]. To determine the effect of Zfp281 binding to this site we restored the mouse site (ctggtag to cCTgCag) by site-directed mutagenesis of the human P1. Luciferase reporter assay showed that restoring the mouse Zfp281 site enhanced the luciferase expression by 35% over the wild-type human P1. To verify Zfp281 binding to the restored mouse site in human P1 we performed EMSAs. DNA fragments containing either the wild-type human P1 or a 3 basepair alteration that restores the mouse Zfp281 binding footprint were synthesized and radiolabeled for use as EMSA probes (Figure 5B). Incubation of the probes with CJ7 nuclear extracts resulted in a shift with the probe harboring a restored mouse Zfp281 binding site and this was not observed with the wild-type human P1 probe. EMSA in presence of excess unlabeled oligonucleotides showed that while the mouse Zfp281 binding-site restored probe was able to compete off the binding, the wild-type probe failed to do so. These results demonstrate that the Zfp281 binding site in human P1 is lost during the course of mammalian evolution and that this contributes to a dampening of *NANOG* P1 activation.

## Discussion

The unique self-renewal and pluripotency characteristics of ESCs are regulated by a multi-layered network of post-transcriptional and post-translational modifications [51]. Alternate splicing augments this regulatory control by increasing transcriptome and proteome diversity from a single gene locus. The resulting additional protein isoforms may contribute to the maintenance of the ESC state or commitment to different lineage specification by modifying the protein interaction network or by localizing to different compartment of the cell. Alternately, changes in non-coding sequences may alter post-transcriptional regulation. Alternative splicing points to alternate promoter selection and the activation or inhibition of different promoters for the same gene may impact lineage commitment and differentiation. The core pluripotency factor *OCT4* is known code for multiple transcripts and protein variants, out of which only *OCT4A* is implicated for pluripotency of ESCs, whereas the other versions, *OCT4B* and *OCT4B1* are expressed in more differentiated cell types [52,53]. Our previous studies in mouse ESCs revealed multiple transcripts of *Nanog* capable of coding for two additional protein isoforms with differential capacities for maintaining a pluripotency gene signature [23]. In the present study, we verified the transcription of *NANOG* from a novel upstream exon in human ESCs and identified a strong promoter upstream (P2) responsible for transcription initiating from this exon. Although there was a more robust DNAseI HS regulatory footprint at P2, we observed that there was a decreased enrichment of Oct4 and p300 at this region that might affect assembly of a robust RNA transcriptional complex. Alternatively, transcripts may be produced with comparable strength at both P1 and P2, but diminished elongation of transcripts from P2 resulting from a less processive RNA polymerase complex or due to post-transcriptional regulatory mechanisms might contribute to the lower levels of fully processed transcripts produced from this alternate promoter. We also determined that Nanog employs a positive autoregulatory mechanism to control its own downstream promoter (P1).

**Figure 6:**
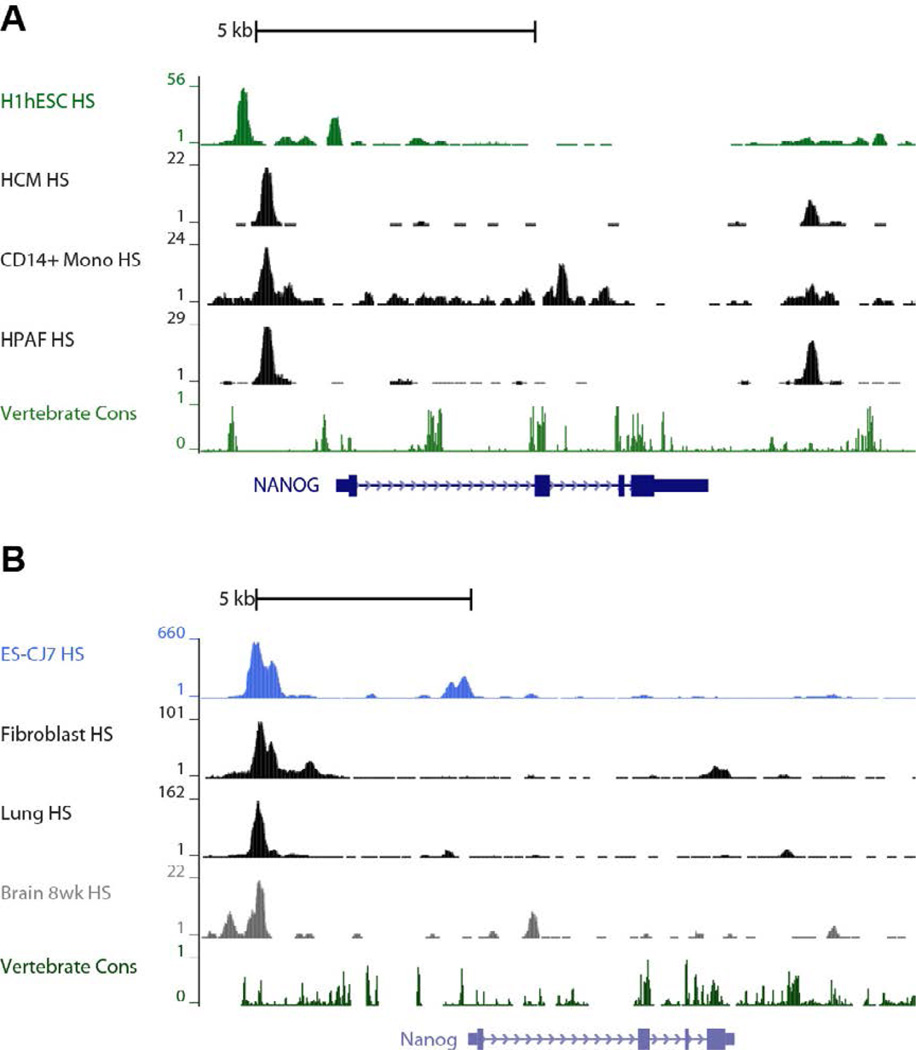
NANOG promoter P2 sequence conservation and DNAseI HS at the NANOG locus in different tissues (A) *NANOG* gene structure is shown at the bottom. DNAseI HS peaks in H1 ESCs, human cardiomyocytes (HCM), CD14 monocytes and human pancreatic adenocarcinoma cells (HPAF) showing the active *cis*-regulation mark at the upstream P2 promoter persists in adult differentiated cells, whereas the HS mark observed in the ESCs at the proximal P1 promoter is extinguished in the adult cells. (B) A similar persistence of the HS peak at P2 is observed in mouse adult cells while the downstream P1 peak is extinguished.

The transcription factor Nanog functions collaboratively with the other core pluripotency factors Oct4 and Sox2 to govern the maintenance of pluripotency. Even though Nanog was originally shown to be critical largely for repressing primitive endoderm formation [25], work by others implies that it is responsible for repressing genes essential for germ layer specification in the embryo proper as well [54]. Using a system that enabled the controlled loss of Nanog function in ESCs, we recently demonstrated that Nanog functions as a global repressor of critical genes that underlie function of the early mesodermal, endodermal and ectodermal germ layer compartments, as well as primitive endoderm [23]. Nanog remains functionally unique among the core pluripotency triad for this global gene repression capacity. We were intrigued by this phenomenon and wished to determine if regulatory elements positioned near the Nanog gene might provide mechanistic insight on how Nanog represses genes responsible for lineage specification. By analyzing HS that marked the Nanog promoters in adult cells and organs of mice and humans, we revealed strong regulatory element footprints in tissues (Figure 6). Intriguingly, when a 5712 bp DNA cassette containing both Nanog promoters is inserted into the beta globin locus that is permissive for expression only in erythroid cells, DNA methylation levels reveal that both regulatory elements remain active in ESCs [55]. This suggests that Nanog regulatory elements are sufficient to remodel a normally repressive chromatin domain and make it amenable for gene expression. In contrast, upon differentiation into neural progenitor cells, the upstream promoter region remains unmethylated and active but the downstream promoter is completely extinguished. This implies that the upstream alternate promoter harbors regulatory elements that enable it to remain permissive for gene expression in pluripotent and adult cells. Previous work has suggested that genes of certain cellular lineages may be marked in pluripotent cells by a histone signature that poises gene expression to be turned on later during lineage specification in a well-defined spatial and temporal manner [56]. Conversely, this Nanog regulatory element may serve as a platform to mark a potent repression footprint that ensures that certain Nanog protein variant(s) are not expressed in adult lineages. Compelling hints have suggested that Nanog may function in adult tissues [57–61]. For this reason, we cannot rule out the possibility that Nanog alternative transcripts expressed from the upstream promoter may encode proteins that function to regulate tissues in post-embryonic stem and progenitor cell populations.

An alignment of the most active human P2 region (−1788 to −1737, Figure 3B) showed that the sequence is well conserved among vertebrates. However *NANOG* P1 and P2 are narrowing a genomic gap during evolution as the distance of 5 kb in the mouse has gradually contracted down to 1.8 kb in the human gene locus. The sequence analysis of this region in TRANSFAC predicted binding sites of potent components of the basal transcriptional machinery. The inclusion of TFIID, CBP, CEBPA and NF-1 (Figure 3A) binding footprints within this short sequence might explain the significant strength of this small promoter in comparison to the proximal P1 promoter (Figure 3C). Two ATTA/C sites predicting NANOG binding motifs are also present in this short sequence. The upstream site is on the sense strand, the downstream site lies on the antisense strand, and this Nanog tandem likely confers strength upon this promoter. One of these sequences has also been experimentally validated as a Nanog binding site in mouse ESCs [15].

While NANOG binding to the *NANOG* proximal promoter has been demonstrated [41], the regulatory effect of this binding has not been analyzed previously. Utilizing site-directed mutagenesis and EMSAs we identified the Nanog binding sites in the proximal promoter and showed that Nanog contributes to the activation of its own promoter. Nanog is known to exist as a homodimer in ESCs [36]. Given the proximity of the two Nanog binding motifs in P1, it is possible that Nanog binds to these sites through a monomeric or homodimeric configuration.

The human P1 resulted in expression of neomycin in our stable transfection assay; however, the promoter strength measured by this assay was significantly weaker in comparison to the P2 minimal promoter (Figure 3C). Additionally, a mouse P1 of near identical size was significantly stronger than the human P1 (unpublished observations). Sequence alignment of the conserved P1 sequences showed that while the Oct4-Sox2 and Nanog binding sites are well conserved in the P1 sequence, a binding site for the pluripotency factor Zfp281 is lost in the human promoter (Figure 4A). Zfp281 has been shown to activate the mouse P1 [49] by binding to this site. Another pluripotency factor Zic3 has also been shown to activate the mouse P1 by binding to the same region [50]. When we restored the human P1 sequence to match the mouse binding site, it resulted in a significant increase in human P1 activity (Figure 4B). Zfp281 is known to interact with Nanog [7,36]. In the absence of Zfp281, Nanog recruitment to the Nanog site in mouse P1 was shown to be significantly diminished in the absence of Zfp281 in ESCs [62]. Perhaps in the absence of an intact Zfp281 site in *NANOG* P1, efficient Nanog recruitment is diminished. As we have shown that Nanog activates P1 activity, less efficient binding could explain its loss of strength.

## Conclusion

The discovery of an alternate NANOG promoter that functions in ESCs significantly expands the regulatory repertoire used to control this core pluripotency factor. A comprehensive study of the human *NANOG* locus in human ESCs will be required in the future to reveal the full upstream exon structure and to determine if additional protein variants exist, as suggested by our recent work in mouse ESCs [23]. The existence of a potent alternate promoter positioned at the 5’ end of the gene locus presents an additional mechanism for dampening expression of the predominant NANOG transcript and protein through the production of upstream open reading frames (uORFs) [63]. Alternative transcripts that we discovered in mouse ESCs exhibit loss or gain of large uORFs [23] that frequently serve to sequester the translational apparatus away from downstream coding sequences. Future work will be required to determine if the human upstream *NANOG* promoter serves a dual role in producing novel protein variants and downregulating the predominant NANOG A protein.

## Figures and Tables

**Table 1:** Primers used in the Study For the following promoter primers the attB site sequences are shown in capital letters. Information for EMSA primers can be found in Figure 5.

Usage	Primers Used (5’–3’)	Amplimer size

Promoter-Reporter	hsNgProm −2401-AttB1-F1: ggggACAAGTTTGTACAAAAAAGCAGGCTTAtctctcatgcctttacccaaa	
	hsNgProm −1817-AttB1-F1: ggggACAAGTTTGTACAAAAAAGCAGGCTTaaaaatctaaagtcagatagcttcc	
	hsNgProm −1788-AttB1-F1: ggggACAAGTTTGTACAAAAAAGCAGGCTTAcctcaactttattccaattgcttt	
	hsNgProm −1737-AttB2-R1: ggggACCACTTTGTACAAGAAAGCTGGGTAggccgacttactacattcttcg	
	hsNgProm −1702-AttB2-R1: ggggACCACTTTGTACAAGAAAGCTGGGTAgtgaaagaccaaagggaagg	
	hsNgProm −258-AttB1-F1: ggggACAAGTTTGTACAAAAAAGCAGGCTTAtcccattcctgttgaaccat	
	hsNgProm +34-AttB2-R1: ggggACCACTTTGTACAAGAAAGCTGGGTAaacgttaaaatcctggagtctct	
	Hoxb6 Prom-AttB1-F1: ggggACAAGTTTGTACAAAAAAGCAGGCTTaggttgataggtttgtgcgc	
	Hoxb6 Prom-AttB2-R1: ggggACCACTTTGTACAAGAAAGCTGGGTAccgggtttatgatttgttgtgt	

qRT-PCR	NANOG F3: tgtgggcctgaagaaaactatc	111 bp
	NANOG R3: gctgtcctgaataagcagatcc	
	NANOG F1: tccctttggtctttcactcc	55 bp
	NANOG R1: ctccctgtcccattgtgtct	
	NANOG F2: tatgcaaagacccccttctg	58 bp
	NANOG R2: gctctccaaagggcaggta	
	GAPDH F1: tgggtgtgaaccatgagaagta	125 bp
	GAPDH R1: gagtccttccacgataccaaag	
